# Case Report: “Stent-within-a-Stent” technique after telescopic flow diverter reconstruction for basilar artery aneurysm with proximal stenosis

**DOI:** 10.3389/fsurg.2025.1603876

**Published:** 2025-09-16

**Authors:** Iroda Mammadinova, Chingiz Nurimanov, Nurtay Nurakay, Karashash Menlibayeva, Serik Akshulakov, Yerbol Makhambetov

**Affiliations:** ^1^ Department of Vascular and Functional Neurosurgery, National Center of Neurosurgery, Astana, Kazakhstan; ^2^ Department of Population Health Sciences, Faculty of Life Sciences and Medicine, King’s College London, London, United Kingdom

**Keywords:** basilar artery aneurysm, complex aneurysm, preaneurysmal stenosis, telescopic stenting, stent-within-a-stent

## Abstract

**Background:**

Complex basilar artery aneurysms are challenging to treat due to their deep anatomical location and proximity to critical perforating arteries, as well as their frequent fusiform morphology and association with long-segment stenosis or atherosclerotic changes. Endovascular flow diversion has become an important option; however, its use in large and fusiform basilar artery aneurysms is complicated by the risks of device malapposition, migration, and incomplete occlusion.

**Case presentation:**

We report a case of a large basilar artery aneurysm with severe pre-aneurysmal stenosis treated using a telescopic flow-diverter strategy. Balloon angioplasty prior to stent deployment was performed to optimize vessel diameter, followed by the sequential distal-to- proximal placement of overlapping flow diverters. Post-procedural angiography revealed device instability, necessitating the deployment of an additional flow diverter in a “stent-within-a-stent” configuration to improve wall apposition and prevent migration. This approach resulted in sustained aneurysm thrombosis and long-term vessel patency.

**Conclusion:**

This case illustrates the technical challenges of managing complex basilar artery aneurysms and emphasizes the role of adjunctive stenting in stabilizing flow diverters. Carefully individualized endovascular strategies that account for fusiform morphology, long-segment arterial involvement, atherosclerotic changes, and the perforator-rich environment, together with technical challenges such as the need for telescopic reconstruction or adjunctive balloon angioplasty, are essential for enhancing procedural safety and achieving durable aneurysm occlusion in high-risk patients.

## 1 Introduction

The management of complex basilar artery aneurysms (BAA) is among the most difficult challenges in neurointerventions, as both microsurgical and endovascular approaches are associated with substantial risks. These aneurysms are challenging to manage because of their deep anatomical location and proximity to critical perforating arteries, compounded by factors such as fusiform morphology and proximal stenosis, which increase the risk of thromboembolic events and limit device options (1). Historically, surgical clipping and deconstructive techniques often required parent vessel sacrifice with uncertain collateral support and high morbidity. The introduction of flow diverters (FD) has transformed aneurysm management by enabling parent artery preservation and promoting intra-aneurysmal thrombosis (2, 3). While FDs are highly effective in the anterior circulation, their use in the posterior circulation, particularly for fusiform and large BAAs, remains technically demanding (4). In such cases, a single FD may not achieve sufficient effect, potentially leading to persistent filling, delayed thrombosis, or even rupture. Long-segment reconstructions are also technically complex and may increase the risk of malapposition, incomplete expansion, or migration (5). To address these challenges, telescopic placement of multiple FDs has been developed to enhance flow modification and improve construct stability (6).

In this report, we describe a case of a complex BAA with pre-aneurysmal stenosis managed in a single session using balloon angioplasty followed by telescopic FD reconstruction. The procedure was complicated by instability at the junction of two telescopically deployed stents, resulting in a potential risk of migration into the aneurysm. This case underscores the evolving role of adjunctive stenting in posterior circulation aneurysm management and highlights the importance of adaptive endovascular strategies for high-risk vascular reconstructions.

## 2 Case description

### 2.1 Clinical details

A patient in his fifties presented with a history of ischemic stroke in the posterior cerebral artery territory 2 months prior. The patient exhibited cerebellar ataxia, but showed no other clinically significant neurological abnormalities.

### 2.2 Neuroimaging

**Figure 1:**
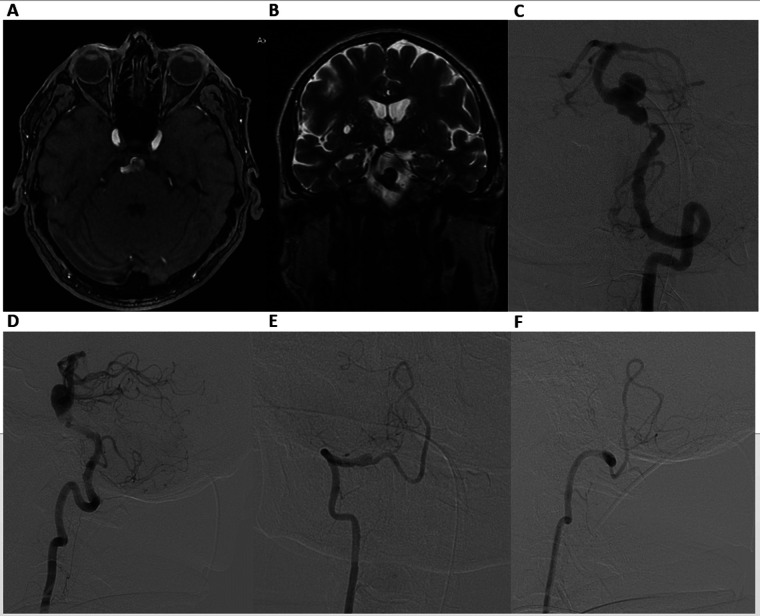
Axial time-of-flight (TOF) MR angiography **(A)**, coronal T2-weighted MRI **(B)**, demonstrating a basilar artery aneurysm. Digital Subtraction Angiography. Frontal **(C)** and lateral **(D)** views of the left vertebral artery, showing a wide-neck aneurysm in the inferior third of the basilar artery, accompanied by nearly 90% preaneurysmal stenosis. Frontal **(E)** and lateral **(F)** views of the right vertebral artery, revealing V4 segment occlusion distal to the right posterior inferior cerebellar artery.

MRI and MR angiography revealed a partially thrombosed BAA (Figures 1A,B). Digital Subtraction Angiography (DSA) confirmed the aneurysm (13 mm × 11 mm) with a wide neck (12 mm) in the inferior third of the BA, accompanied by approximately 90% pre-aneurysmal stenosis (0.5 mm at the level of stenosis), posing a high risk for hemodynamic compromise and potential thromboembolic events (Figures 1C,D). DSA also revealed V4 segment occlusion distal to the right posterior inferior cerebellar artery (Figures 1E,F).

### 2.3 Surgical technique

Given the presence of a symptomatic BAA with associated stenosis, endovascular treatment was planned. The procedure was performed under general anesthesia after the patient had been premedicated with dual antiplatelet therapy and intraoperative anticoagulation to reduce thromboembolic risk. The patient had a history of ischemic heart disease and had been taking Aspirin 100 mg daily for 1 year. For the intervention, Aspirin 100 mg was continued, Ticagrelor 180 mg was administered 2 h before the procedure, and 5,000 i.u. of intravenous heparin was given intraoperatively prior to stent deployment.

The patient initially underwent balloon angioplasty to dilate the preaneurysmal stenosis and restore adequate blood flow in the BA, optimizing conditions for subsequent stent deployment.

**Figure 2:**
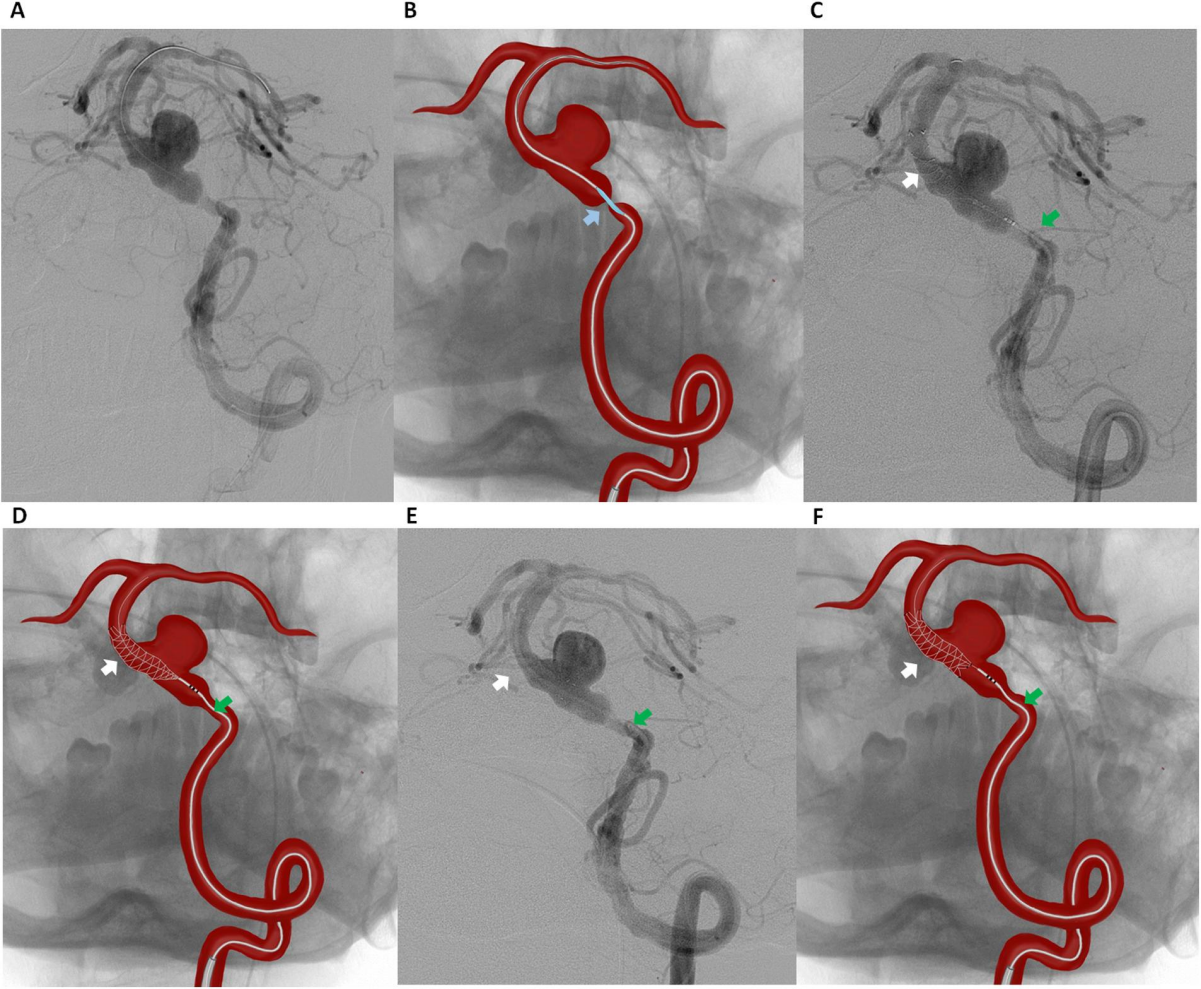
Digital subtraction angiography **(A)** and schematic illustration **(B)** of the left vertebral artery showing balloon angioplasty of the preaneurysmal critical stenosis of the basilar artery (blue arrow indicating the accuforce 3.5 mm × 15 mm balloon catheter). Digital Subtraction Angiography **(C)** and schematic illustration **(D)** showing flow diverter placement from the middle third of the basilar artery (white arrow indicating the FredX 4.00 mm × 20 mm flow diverter, green arrow indicating the Headway-027 microcatheter). Digital Subtraction Angiography **(E)** and schematic illustration **(F)** of the left vertebral artery after stent (FredX 4.00 mm × 20 mm) deployment.

A 6F guiding catheter was advanced into the left vertebral artery via a transfemoral approach. Under roadmap guidance, a Headway 027 microcatheter was navigated across the stenotic segment of the basilar artery over a 0.014-inch Asahi Chikai microwire. A 3.5 mm × 15 mm Accuforce balloon catheter was carefully positioned across the preaneurysmal stenosis. The balloon was inflated up to 8–10 atm under fluoroscopic control to gradually expand the stenotic segment while minimizing the risk of vessel injury or plaque disruption (Figures 2A,B). After maintaining inflation for a few seconds, the balloon was deflated and carefully withdrawn. Immediate DSA confirmed luminal gain in the stenotic segment.

Following successful angioplasty, our initial plan was to deploy a FD for aneurysm embolization. However, FD were not available in our setting at the time. Therefore, a telescopic stent deployment strategy was chosen to ensure complete coverage of the aneurysm neck. The parent vessel measured 4.05 mm proximally and 4.12 mm distally to the aneurysm neck. A FredX 4.00 mm × 20 mm FD was carefully navigated and deployed from the middle third of the basilar artery using a Headway-027 microcatheter, ensuring optimal coverage of the aneurysm neck while aiming to maintain perforator patency (Figures 2C–F).

**Figure 3:**
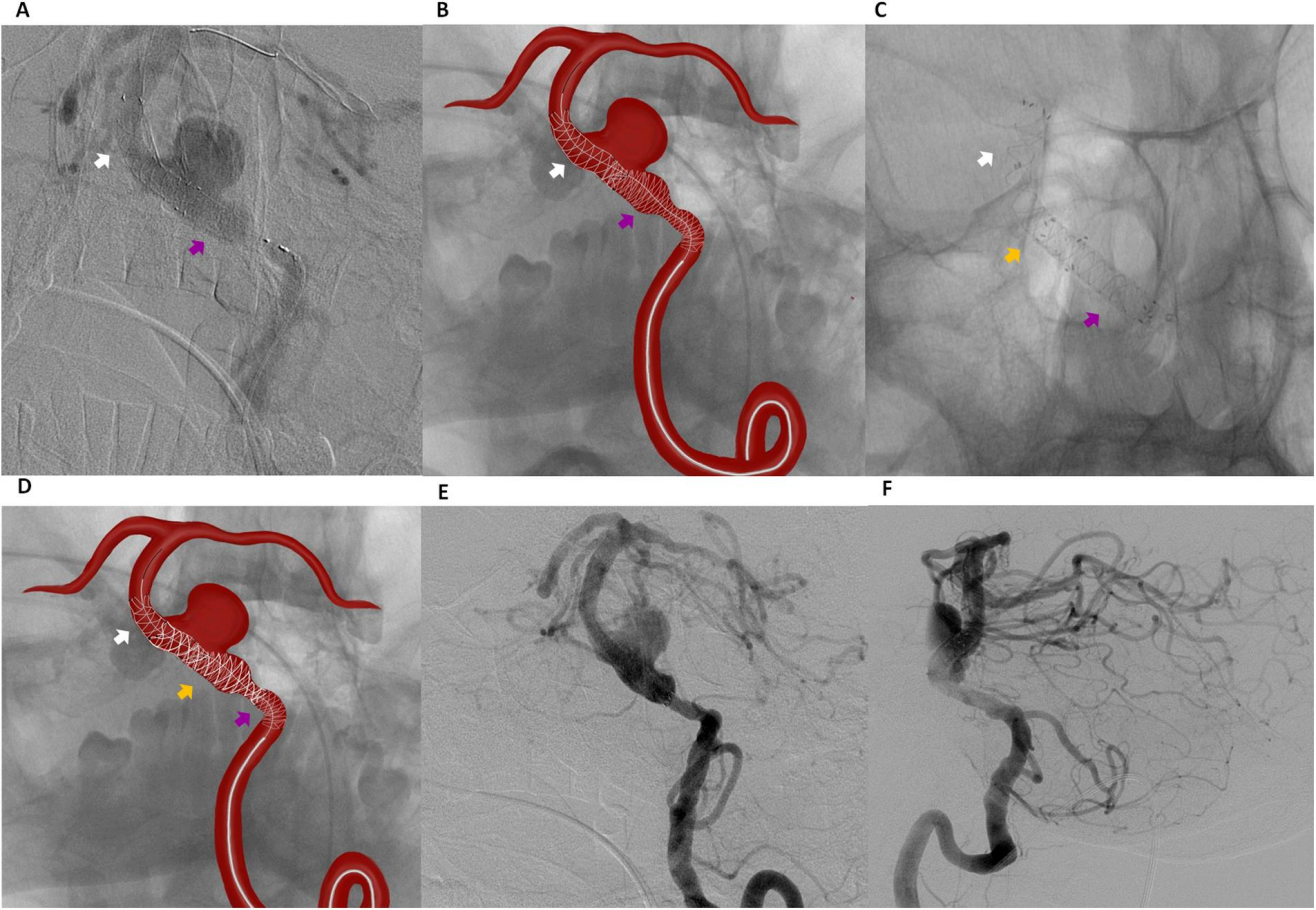
Digital subtraction angiography **(A)** and schematic illustration **(B)** showing telescopic placement of a second flow diverter (pipeline 4.00 mm × 20 mm) extending from the middle portion of the previously placed fredX 4.00 mm × 20 mm stent (white arrow indicating the fredX 4.00 mm × 20 mm flow diverter, violet arrow indicating the pipeline 4.00 mm × 20 mm). Digital Subtraction Angiography **(C)** and schematic illustration **(D)** showing a “Stent-within-a-Stent” placement of a third flow diverter, FredX 3.50 mm × 22 mm (orange arrow), extending from the middle portion of the previously placed FredX 4.00 mm × 20 mm stent (white arrow) to the middle portion of the previously placed Pipeline 4.00 mm × 20 mm stent (violet arrow). Digital Subtraction Angiography: Frontal **(E)** and lateral **(F)** views of the left vertebral artery immediately after surgery.

Given the occlusion of the contralateral vertebral artery and the absence of collateral circulation via the communicating arteries, a second FD with coverage of the stenotic segment was deployed. A second Pipeline 4.00 mm × 20 mm FD was implanted in a telescopic fashion, extending from the middle portion of the previously placed FredX 4.00 mm × 20 mm stent and involving the stenotic segment (Figures 3A,B).

Post-deployment DSA demonstrated instability due to shortening of the first two 4.0 mm FDs. The first stent was undersized relative to the vessel diameter, resulting in shortening and covering approximately 75% of the aneurysm neck. The second stent 4.0 mm stent, placed in the widest vessel segment, also demonstrated shortening. To stabilize the construct and prevent potential migration into the aneurysm, a third 3.5 mm FD (FredX 3.50 mm × 22 mm) was deployed in a stent-within-a-stent configuration (Figures 3C,D) using a Headway-027 microcatheter navigated through the previously placed stents under roadmap guidance.

Post-procedural angiography confirmed successful reconstruction of the BA. DSA immediately after the procedure demonstrated delayed contrast filling of the aneurysm (O’Kelly-Marotta grade B) without evidence of endoleak or significant in-stent stenosis (Figures 3E,F). The patient was discharged on the second postoperative day without neurological deficits. At discharge, dual antiplatelet therapy was prescribed, consisting of Ticagrelor (90 mg twice daily) for 6 months and Aspirin (100 mg daily) for long-term therapy. The patient was already receiving Atorvastatin 20 mg daily, which had been prescribed by the cardiologist prior to admission.

### 2.4 Follow-up

**Figure 4:**
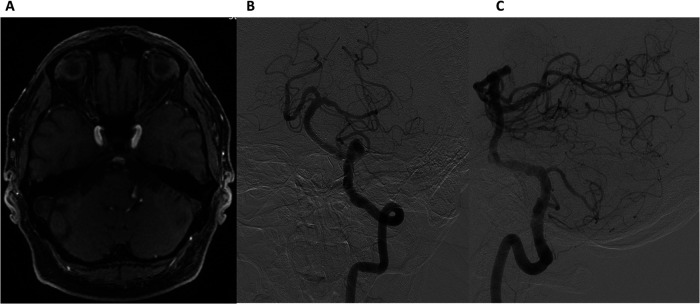
Follow-up at 3 months. Axial time-of-flight (TOF) MR angiography **(A)** showing aneurysm thrombosis. Digital Subtraction Angiography: Frontal **(B)** and lateral **(C)** views of the left vertebral artery demonstrating non-significant residual stenosis in the basilar artery and the absence of flow in the aneurysm.

At the 3-month follow-up, the patient reported complete resolution of vertigo, with no new neurological symptoms. Axial TOF MR angiography (Figure 4A) demonstrated thrombosis of the aneurysm, confirming successful flow diversion. DSA (Figures 4B,C) demonstrated the absence of flow within the aneurysm and non-significant residual stenosis (approximately 20%) in the BA. These findings indicate successful flow diversion, effective aneurysm occlusion, and preserved vascular patency. At follow-up, Ticagrelor (90 mg twice daily) was discontinued at 6 months, while Aspirin (100 mg daily) was continued as long-term therapy.

## 3 Discussion

Endovascular techniques for complex BAAs have advanced significantly, with flow diversion becoming a key strategy for managing large and fusiform aneurysms in anatomically challenging locations (5, 7–9). In this context, the potential link between proximal stenosis and aneurysm formation remains an important consideration, as stenotic segments can induce hemodynamic alterations that contribute to vascular remodeling. These changes may increase wall stress, predisposing the vessel to progressive dilation and, over time, aneurysm development (1).

Unlike conventional stent-assisted coiling, which relies on mechanical occlusion often fails to reduce the mass effect in large aneurysms (10), FD promotes intra-aneurysmal thrombosis while reconstructing the parent artery. Additionally, stent-assisted coiling may not effectively reduce the mass effect in large aneurysms, as the aneurysm sac remains packed with coils, potentially exerting continued pressure on adjacent brainstem structures (7, 11–14). In contrast, FD treatment has been shown to achieve favorable volumetric reduction and aneurysm sac shrinkage, often resulting in radiologically confirmed reversal of mass effect and clinical improvement in most cases (15).

However, in large and fusiform BAAs, a single FD may not provide adequate coverage. In this case, the landing zones were limited by the long-segment aneurysm and proximal stenosis, leaving insufficient healthy vessel for stable anchoring and thereby increasing the risk of malapposition, incomplete coverage, and device instability (5, 16, 17).

Given these challenges, telescopic flow diversion, or double-stent embolization, involves deploying multiple overlapping FDs in a staged or sequential manner to achieve durable vessel reconstruction. In our case, the stents were deployed in a distal-to-proximal sequence. This approach facilitated easier navigation and ensured stable anchoring in the distal healthy segment before extending coverage proximally across the aneurysm. Conversely, a proximal-to-distal deployment strategy may in some cases provide stronger anchorage at the vertebral side and reduce the risk of foreshortening, though it is technically more demanding and increases the risk of distal device migration (16, 18, 19).

In wide-neck complex aneurysms, telescopic stenting offers several advantages over single FD deployment (2, 16, 20). First, it increases metal coverage, enhancing flow diversion and accelerating aneurysm occlusion. Second, it improves construct stability in long-segment or fusiform aneurysms by reducing the risk of device migration or shortening. Third, when the initial FD fails to fully expand due to vascular tortuosity or poor wall apposition, an additional FD can optimize device conformation and adherence, minimizing the risk of residual aneurysm filling.

A key factor in our treatment strategy was the presence of proximal preaneurysmal stenosis, which significantly altered basilar artery hemodynamics and influenced our approach to endovascular reconstruction. This stenosis introduced several challenges, including an elevated risk of postprocedural thromboembolic complications and potential inadequate device expansion (21). To address this, we performed balloon angioplasty before FD deployment to optimize vessel diameter, ensuring proper FD expansion and reducing the risk of malapposition (16). The angioplasty was carefully executed to minimize the risk of endothelial injury or dissection.

Initially, we chose a telescopic FD approach over a single-device strategy. However, despite its theoretical advantages, we encountered FD instability, necessitating additional intervention to secure the construct and prevent stent malapposition (16). Instability was likely related to a combination of FD shortening after expansion, a known issue in long-segment reconstructions, and the presence of proximal stenosis, which further compromised device anchoring and contributed to the risk of migration.

To stabilize the construct, we employed a stent-within-a-stent technique, deploying an additional FD to enhance metal coverage, improve FD wall apposition, and provide mechanical support (18, 19). This approach is supported by previous studies showing that additional stenting provides several important benefits. First, it enhances metal coverage, increasing construct density and further reducing inflow into the aneurysm, thereby accelerating thrombosis. Second, it improves device wall apposition, which is particularly crucial in large-caliber vessels where incomplete FD expansion may lead to endoleaks and persistent aneurysm perfusion. Third, it stabilizes the flow diverters, reducing the risk of migration or displacement—a well-documented concern in basilar artery reconstructions due to high-flow dynamics and the frequent need for long-segment coverage.

Despite its advantages, this approach carries certain risks, including an increased metal burden that can elevate thrombogenicity and necessitate prolonged dual antiplatelet therapy, thereby increasing the risk of hemorrhagic complications. Moreover, overlapping stents may lead to flow stagnation in critical perforators, potentially heightening the risk of ischemic events. Stent rigidity and vessel wall irritation could also contribute to delayed in-stent stenosis or endothelial dysfunction. Another limitation of telescopic deployment is the substantially increased procedural cost, primarily due to the use of multiple devices.

Oversized stenting has been considered as an alternative to telescopic flow diversion in cases of large and fusiform aneurysms. By deploying a larger-diameter FD, operators aim to achieve sufficient metal coverage while minimizing the need for multiple overlapping devices. However, this approach presents several challenges. Studies have shown that excessive oversizing can reduce the diversion effect by altering the porosity of the device, leading to suboptimal aneurysm occlusion (22). Additionally, oversizing has been associated with an increased risk of in-stent stenosis, which may compromise long-term vessel patency and necessitate further intervention (23). Another concern is the difficulty in achieving adequate wall apposition in cases of significant size mismatch, increasing the risk of device migration and malapposition, particularly in tortuous vascular anatomy (24).

Previous studies have shown that triple stent therapy can be more effective than single or double stenting while achieving a metal coverage rate comparable to that of flow diverters (6). As a result, it remains a viable treatment option for complex posterior circulation aneurysms. The development of long-length flow diverters may offer a promising alternative to multiple stenting, improving construct stability while reducing procedural complexity and metal burden in complex aneurysm treatment.

## 4 Conclusion

The management of complex BAAs requires carefully tailored endovascular strategies to achieve durable reconstruction and effective flow diversion. This case illustrates the value of telescopic stenting and adjunctive techniques in stabilizing the construct and overcoming device-related challenges. However, safety considerations must also be emphasized: implanting multiple FDs in perforator-rich territories such as the BA carries inherent risks, including ischemic complications, increased thrombogenicity, and the need for prolonged dual antiplatelet therapy. Individualized patient selection and meticulous procedural planning are therefore essential to balance efficacy with safety in high-risk aneurysm reconstructions.

## Data Availability

The raw data supporting the conclusions of this article will be made available by the authors, without undue reservation.
